# Schools of public health in low and middle-income countries: an imperative investment for improving the health of populations?

**DOI:** 10.1186/s12889-016-3616-6

**Published:** 2016-09-07

**Authors:** Fauziah Rabbani, Leah Shipton, Franklin White, Iman Nuwayhid, Leslie London, Abdul Ghaffar, Bui Thi Thu Ha, Göran Tomson, Rajiv Rimal, Anwar Islam, Amirhossein Takian, Samuel Wong, Shehla Zaidi, Kausar Khan, Rozina Karmaliani, Imran Naeem Abbasi, Farhat Abbas

**Affiliations:** 1 Department of Community Health Sciences, Aga Khan University, Karachi, Pakistan; 2 Pacific Health & Development Sciences Inc., Victoria, Canada; 3 Faculty of Health Sciences, American University of Beirut, Beirut, Lebanon; 4 Division Public Health Medicine, School of Public Health and Family Medicine University of Cape Town, Cape Town, South Africa; 5 Alliance for Health Policy and Systems Research, WHO, Geneva, Switzerland; 6 Hanoi School of Public Health, Giang Vo, Ba Dinh, Hanoi, Vietnam; 7 Depts LIME & PHS, Karolinska Institutet Stockholm, Stockholm, Sweden; 8 Department of Prevention and Community Health, George Washington University School of Public Health and Health Services, Washington, USA; 9 School of Health Policy and Management, York University, Toronto, Ontario Canada; 10 Department of Global Health & Sustainable Development, School of Public Health-Tehran University of Medical Sciences, Tehran, Iran; 11 JC School of Public Health and Primary Care, Faculty of Medicine, The Chinese University of Hong Kong, Sha Tin, Hong Kong; 12 School of Nursing & Midwifery and Department of Community Health Sciences, Aga Khan University, Karachi, Pakistan; 13 Medical College, Aga Khan University, Karachi, Pakistan

**Keywords:** Schools of public health, Low and middle income countries, Universal health coverage, Social determinants of health, Healthcare, Public health education, Health research, Policy development, Collaboration, Partnerships

## Abstract

**Background:**

Public health has multicultural origins. By the close of the nineteenth century, Schools of Public Health (SPHs) began to emerge in western countries in response to major contemporary public health challenges. The Flexner Report (1910) emphasized the centrality of preventive medicine, sanitation, and public health measures in health professional education. The Alma Ata Declaration on Primary Health Care (PHC) in 1978 was a critical milestone, especially for low and middle-income countries (LMICs), conceptualizing a close working relationship between PHC and public health measures. The Commission on Social Determinants of Health (2005–2008) strengthened the case for SPHs in LMICs as key stakeholders in efforts to reduce global health inequities. This scoping review groups text into public health challenges faced by LMICs and the role of SPHs in addressing these challenges.

**Main text:**

The challenges faced by LMICs include rapid urbanization, environmental degradation, unfair terms of global trade, limited capacity for equitable growth, mass displacements associated with conflicts and natural disasters, and universal health coverage. Poor governance and externally imposed donor policies and agendas, further strain the fragile health systems of LMICs faced with epidemiological transition. Moreover barriers to education and research imposed by limited resources, political and economic instability, and unbalanced partnerships additionally aggravate the crisis. To address these contextual challenges effectively, SPHs are offering broad based health professional education, conducting multidisciplinary population based research and fostering collaborative partnerships. SPHs are also looked upon as the key drivers to achieve sustainable development goals (SDGs).

**Conclusion:**

SPHs in LMICs can contribute to overcoming several public health challenges being faced by LMICs, including achieving SDGs. Most importantly they can develop cadres of competent and well-motivated public health professionals: educators, practitioners and researchers who ask questions that address fundamental health determinants, seek solutions as agents of change within their mandates, provide specific services and serve as advocates for multilevel partnerships. Funding support, human resources, and agency are unfortunately often limited or curtailed in LMICs, and this requires constructive collaboration between LMICs and counterpart institutions from high income countries.

## Background

### Historical evolution of public health

Public health is described as the art and science of promoting and protecting good health, preventing disease, disability and premature death, restoring good health when it is impaired by disease or injury, and maximizing quality of life [1]. Its historical origins are ancient, among the earliest evidence being the Indus Valley Harappan civilization (3500–1500 BCE) water and sanitation systems, and the Code of Hammurabi (circa 2000 BCE) of ancient Mesopotamia [2]. Greek physician Hippocrates (400 BCE) noted that infected people did not become re-infected from smallpox. This led to experiments in China (circa 1000 CE) giving rise to vaccinology. European scholars such as Agricola (1494–1555) recorded the occupational hazards of mining and smelting. John Snow (1813–1858) and TK Takaki (1858–1890) applied epidemiologic approaches to practical public health interventions in order to tackle Cholera and Beriberi, while Rudolf Virchow (1821–1902) promoted “social medicine” and the need for health supporting social policies, especially in impoverished and unjust settings [3, 4]. Such landmark observations from several regions remind us that public health is not a uniquely western concept.

By the close of the nineteenth century, SPHs began to emerge in western countries, such as the London School of Hygiene and Tropical Medicine (established 1899) and the Tulane University School of Hygiene and Tropical Medicine (established 1912) [5, 6]. Concurrently, the philosophy of social medicine was embodied in the United States Flexner Report (1910), which noted “the overwhelming importance of preventive medicine, sanitation, and public health” in the context of medical education [7]. Conceptualization of independent SPHs in the United States subsequently gained traction from the 1915 Welch-Rose Report [8]. The Rockefeller Foundation mobilized this model worldwide through funding and promotional investments, with Johns Hopkins School of Public Health receiving its first endowment in 1916 [8, 9]. In tandem, Virchow’s ‘social medicine’ philosophy grew influence, notably in Schools of Medicine and SPHs in Latin America [10]. Following formation in 1948, the World Health Organization (WHO) endorsed public health education, thus encouraging development of SPHs [9].

The Alma Ata Declaration on PHC in 1978 is an important contemporary milestone for public health. Conceptualizing a close relationship between PHC and public health measures [11], its great promise was unfulfilled, especially for LMICs where donor countries drove development assistance priorities with disease-specific initiatives [12]. Consequently, the role of medical care was well appreciated while health policy analysts from LMICs, ahead of their high-income country (HIC) counterparts in attempting to bring integrated thinking to the development of health systems [13], enjoyed little support. The Declaration’s slogan, ‘health for all by the year 2000,’ was taken up at the turn of the millennium as an opportunity to examine what went wrong with this noble goal, especially the failure to operationalize it. In particular, numerous critics drew attention to serious inequity in PHC delivery and management [14, 15].

The WHO Commission on Social Determinants of Health (2005–2008) drew attention to extreme health inequities between and within countries; its final report contained recommendations to improve daily living conditions, tackle the inequitable distribution of power, money, and resources, and to assess the impacts of interventions [16]. The Commission’s findings reinforced the case to strengthen Schools of Public Health (SPHs) in LMICs as key stakeholders in efforts to reduce global health inequities.

Subsequent frameworks have been developed to educate health professionals about addressing social determinants [17]. Given the global development of health technologies and increasing attention to financial health protection, LMICs are adopting strategies for universal health coverage (UHC); Iran’s experience with urban PHC [18], and Pakistan’s PHC experience utilizing lady health workers provide relevant examples [19].

### Methodology

Keeping in view this historical evolution, we build our thesis on the collective experience of the co-authors and a scoping review conducted by co-author LS to explore the available literature on the public health challenges being faced by LMICs and role of SPHs in responding to these challenges.

A preliminary search in PubMed using key terms ‘schools of public health’ and ‘low and middle-income countries’ revealed public health education, public health research, and partnerships to be consistent topics. We subsequently conducted three separate searches of PubMed and Medline combining key terms ‘schools of public health’ and ‘low and middle-income countries’ with ‘public health education,’ ‘public health research,’ and ‘partnerships.’ Records yielded from each database search had titles screened for topic relevance, and selected records were then assessed for inclusion criteria: a) SPHs in LMICs and b) public health education, research, and/or partnerships.

Three hundred thirty-six articles of the 3233 database hits were retained. These articles were assessed for inclusion criteria, and 46 articles were included. Data extraction and analysis confirmed public health education, public health research, and partnerships as themes that reflect the role of SPHs in LMICs. We supplemented the scoping review with reference searching and grey literature to identify additionally relevant articles and explored examples of SPHs in LMICs.

This paper synthesizes the literature to first detail the public health challenges faced by LMICs, followed by ways SPHs provide a direction to address these challenges. In this paper we adopt an all-encompassing definition of Schools of Public Health as post-secondary departments, institutions, or centers organizing the provision of formative and lifelong public health education for professional development through competency-based curricula, comprehensive public and population health programs, and teaching pedagogies involving inter- and multidisciplinary professionals (including those from administration and management background) and teams in multi-level, inter-sectoral partnerships for collaborative research and development initiatives. We conclude with discussion on key issues to address in the development of SPHs.

## Main text

### Public health challenges being faced by LMICs

LMICs are layered with health burdens rooted in complex political, economic, social, environmental, and demographic realties that shape the functionality of SPHs and their role in addressing population health needs [20]. These challenges include poor health governance and fragile health systems, mass displacements associated with acute and recurrent conflicts and natural disasters, rapid urbanization, environmental degradation, unfair terms of global trade, international financial systems undermining capacity for equitable growth and epidemiological transitions characterized by persistent infectious disease and emerging non-communicable disease burdens [16, 20–23].

Poor health system governance results in population health needs and health spending priorities being demoted, typically in favor of military spending and the interests of political elites with roots in corruption [24–28]. Additionally, restrictive trade agreements with HICs often facilitated by the World Trade Organization and the conditions attached to structural adjustment loans administered to LMICs limit the policy space and capacity of governments, undermining their ability to address health system and population health concerns [29–31]. For example, intellectual property rules in free trade agreements have increased prices of previously affordable generic drugs in LMICs such as Jordan and Guatemala [32], thus compromising the availability and affordability of medicines for their health systems. In addition, multinational corporations exert powerful influences on population health from production and market manipulation to selling of lethal and unhealthy products [33].

Low investment in rural agricultural economies, political instability and conflict, and degrading environmental conditions result in rural to urban movement of economic migrants and mass uprooting of internally displaced persons (IDPs) and refugees who exert a high pressure on the infrastructure and health systems of the country and its neighbors [22, 24, 26, 30–32, 34, 35]. In Syria for example, additional to conflict-driven displacement, thousands of rural Syrians employed in the agricultural sector were forced to migrate to cities in 2006 when a persistent drought coupled with ineffective environmental policies compromised their livelihood [24]. As of January 2016, over 4.5 million registered Syrian refugees have been displaced by conflict, fleeing to neighboring countries of Lebanon, Turkey, Iraq, and Jordan [36]. Although cities offer greater access to improved living standards, social services, employment, and security as compared to rural areas [29, 36], rapid population growth, which is expected to be concentrated in LMICs, pushes services and infrastructure beyond capacity, exposing the populations to overcrowding, water scarcity, inadequate sanitation, food insecurity, poverty and unemployment [24, 37–40].

Non-Communicable Diseases (NCDs) are now the primary cause of death globally, and burgeoning in LMICs. Considering the unprecedented growth of these diseases and their risk factors, in 2011 more than 190 world governments adopted a political declaration and an action plan for reducing premature deaths caused by NCDs by 2025 [37]. In virtual opposition to this accord, transnational corporations use trade and investment agreements to pressure LMICs governments against the implementation of health-protective policies, such as sale restrictions on health-damaging commodities (e.g. tobacco) while capitalizing on unfair trade and investment agreements that make highly processed food and other health-damaging commodities more affordable and accessible to the urban poor [29, 30, 41–43].

SPHs in LMICs have enormous potential to improve health, but not without first overcoming challenges to education and research imposed by scarce resources, antithetical context (e.g. political and economic instability) and unbalanced partnerships [44–52]. From external sources, funding has long been program or disease-specific, thereby limiting the agency of local faculty to focus research on contextually specific health issues with attention to a broader array of determinants and with a multidisciplinary perspective. This traditional funding model fails to nurture the infrastructure, administrative skills, and leadership capacities necessary to empower faculty and staff (e.g. librarians, program coordinators, registrars) to build professional competencies into educational programming and to foster a productive research environment [48, 49, 53].

SPHs can develop *educational programs* that will graduate new cadres of health professionals with appropriate skill sets. Unfortunately, in LMICs this interaction is stunted by severe health worker shortages largely attributable to an inadequate number of educational institutions, compounded by an ongoing ‘brain drain’ [44, 53, 54]. Staff shortages at LMIC SPHs are also a major concern [54], as revealed by the 2008 AfriHealth project findings [45]. Of noticeable comparative absence is public health expertise [53, 55, 56], which may reflect the scarcity of SPHs as compared to nursing and medical schools that have expanded in countries such as Pakistan [10, 57, 58]. There are also gaps in the availability of public health programs that address specific LMIC health issues, as is the case in South Asia. For example, despite under-nutrition being a significant regional issue, South Asia has only one Master of Public Health (MPH) program in Public Health Nutrition [59, 60]. Education is even less accessible for students from rural areas, lower social classes, and minority groups [53].

The Commission on Health Professionals for the 21st Century aggregated “degree-granting public health institutions with medical school departments or subunits offering varying degree titles such as community medicine, preventive medicines, or public health” to identify 467 schools or departments of public health worldwide, approximately one fifth of the number of medical schools [61]. The Commission found 50 % of schools or departments of public health to be in Europe and the Americas, followed by 29 % in Asia, and 21 % in Africa and the Middle East. Low or middle-income Asian countries, with a population of approximately 4.2 billion, have one public health program serving 38 million people, while South America, with a population of 385.7 million has one program per 4.7 million people [6]. It is worth noting how these ratios correspond with the deficits in the global health workforce stated by the 2006 World Health Report, whereby the Americas and Europe combined have two-thirds of the global health workforce for approximately 20 % of the global disease burden while Africa and the Eastern Mediterranean have less than 10 % of the global health workforce to address approximately 33 % of the global disease burden [62].

The responsibilities of SPHs are delivered by different academic public health entities that vary in their structure (e.g. independent schools, units affiliated with government ministries, departments within a medical college, faculty within a university), program options (e.g. undergraduate and graduate degrees, short courses), and curricula (e.g. general public health and specialized disciplines) [6, 61]. Therefore, it is important to note that a department or an institute, of community medicine for example, may be fulfilling the responsibilities of an SPH without the title.

Public health professionals require expertise in public health concepts and context-specific determinants of health as well as the leadership and teamwork qualities to conduct multidisciplinary *research* that identifies, assesses, and addresses health issues with strong social justice and equity values [44, 63]. However, existing curricula often fail to integrate competencies that anticipate and respond to these evolving professional requirements [44, 48, 49, 64]. LMICs with weak research systems are limited in their ability to identify and prioritize population health needs, and in turn, unable to investigate and intervene on their determinants [48, 49, 65–67]. Despite carrying 90 % of the global disease burden, LMICs receive only 10 % of the global expenditure on health research [50]. The majority of LMIC governments do not invest in health research, partly because the public and policymakers are not fully aware of its value [50, 66, 68, 69]. Moreover, foreign funding is often program or disease-specific and arrives in the company of conditions that do not always match local priorities. For example, an assessment of health research projects in Western Cape Province of South Africa found that between 2010 and 2011 foreign donors and the South African government funded 92 and 8 % of projects, respectively [66]. Sixty-five percent of projects funded addressed HIV/AIDS and TB, a research scope that neglects the population’s other priority health issues (e.g. mental health, injury, and nutrition). Research priorities thus fail to translate into action due to varied political interests and unresponsive bureaucracies of the recipient countries [70].

Beyond imposed research agendas, faculty of SPHs in LMICs often work in intellectual isolation with limited career options, low salaries, overburdened workloads, and underdeveloped supervision and mentorship skills [44, 46, 50, 51, 60, 71–73]. Such conditions encourage the growth of and reliance on consultancies amongst academic staff, deter future generations from pursuing research and perpetuate dependence on public health education from HIC SPHs through individually funded programs [46, 49, 50, 74, 75]. The pattern of faculty taking on individual consultancies with international organizations to compensate for low academic salaries can stunt institutional capacity by distracting faculty from academic research, teaching, and mentorship responsibilities as well as perpetuating donor control of research design, data, and publication [76]. Finally, much public health education and research is conducted from units within faculties or departments of medicine, which limits access to resources for training and research in population-oriented studies, narrows the multidisciplinary perspective needed and subordinates the public health perspective to the clinical gaze in the political hierarchies of the institution [45].

*Partnership* between researchers and policymakers is complex, and is often associated with discordance and disconnect [52, 77]. Success of researcher-policymaker partnerships is influenced by each stakeholder’s efforts to understand each other’s priorities. Researchers must be aware of how competing factors inundate policymaking processes.

In global health partnerships, disparities in financial and human resources, and the undervaluation of the skills and contextual knowledge of local resources can inflate inequity [60, 78, 79]. Disparities are also caused by differential access to information, training, funding, networking, and publishing in international scientific journals. The result of such partnerships, which almost invariably exist between SPHs in LMICs and institutions in HICs, is often a power dynamic skewed in favor of HIC partners that can perpetuate a top-down approach and may compromise morale and research outcomes in the LMIC setting. These power dynamics privilege the HIC partners who may have more control over priority setting and data ownership among other processes [80].

### SPHs in LMICs: an avenue for addressing challenges

Recognizing these challenges informs ways that SPHs in LMICs can improve, such that their core values and expertise in prevention and population health, multidisciplinary collaboration, and health systems are supported to effectively address relevant health priorities, reduce health inequities, and improve the overall health of populations.

Since SPHs in LMICs are proximate to the public health workforce and the population health needs to which they respond, these can be a critical avenue for health systems development led by faculty familiar with the local context and challenges [46, 64, 71, 81–84]. They should be able to exercise this advantage through application of the Commission on Health Professionals for the 21st Century recommendations for transformative learning producing change agents “with the status, authority, and ability to promote enlightened transformation in society” [44].

The experience of SPH at Tehran University of Medical Sciences (TUMS) in organizing joint MPH programs for pioneer MD/Pharm D/Diploma in Dentistry students to gain MPH degrees can be mentioned here [63]. Tailor-made international courses on leadership, management and planning of malaria in Eastern Mediterranean region, evolved into a regional curricula in line with four interventions recommended by World Health Report 2008 [85, 86]. Similarly, department of Community Health Sciences (CHS) at Aga Khan University (AKU) conducts regular short training courses in disaster management for fragile health systems, hospital management and implementation research (IR) [47, 87].

SPHs in LMICs can contribute to improvement of public health curricula by defining core competencies that integrate knowledge of local health issues and their context-specific determinants (environmental, social, health systems, technological) as well as apply relevant global concepts to local problems [54, 55, 83, 88, 89]. Such upstream thinking is crucial to refocusing graduate training from a clinical care to population health lens [56, 59, 71, 90–92]. In making this shift, student competencies in interpersonal and public communication, negotiation, management and leadership can be facilitated by integrating faculty mentorship opportunities and multidisciplinary projects with local stakeholders into curricula [44, 48, 83, 90, 93, 94]. Furthermore, public health programs can explore pressing human rights and social justice issues into curricula so that students can also develop values of social responsibility [48, 49, 95]. The Executive Master in Health Care Leadership offered by the Faculty of Health Sciences at the American University of Beirut illustrates how SPHs in LMICs can respond to health system human resource needs with competency-based curricula [96]. Current efforts are underway by the Association of Schools of Public Health in Africa (ASPHA) to harmonize MPH curricula based on agreed regional core competencies in public health [48, 97, 98].

Program diversity is important because offering short courses alongside professional or research-oriented graduate programs allows SPHs in LMICs to address short and long-term human resource requirements for specific health issues (e.g. environmental health, reproductive health) or skill gaps (e.g. epidemiology and biostatistics) [58, 60, 72, 81, 99–102]. Makerere University Kampala School of Public Health, in response to the Ebola epidemic in West Africa, organized a training course on outbreak preparedness and response as part of a multidisciplinary task force [101]. The James P. Grant School of Public Health, Bangladesh, established the Professional Skills Training Centre to facilitate lifelong learning to a national and international audience of health professionals by offering short courses that strengthen competencies in relevant health issues (e.g. urban health and governance, scientific writing for young and mid-level researchers and practitioners) [103].

SPHs organize *public health education* as a broad range of programs, competency-based curriculums, and teaching pedagogies [6, 44]. This breadth allows health professionals to remain active in the workforce while continuing their education [56, 82, 94]. For this reason, the Department of Community Health Sciences at Aga Khan University, Pakistan offers standalone courses for the Master of Science (MSc) in Health Policy and Management program as an alternative to a standard full-time degree structure [104]. The courses train diverse working health professionals (not formally enrolled as MSc students) in the design, implementation, and evaluation of health system programs and policy. They have the option to accumulate credits earned leading to a MSc. In Iran, as health education is under the Ministry of Health and Medical Education (MOHME), SPH at TUMS offers various MPH disciplines, i.e. social determinants of health; health system reforms; health technology assessment; to the employees of the ministry on the basis of ministry’s needs. Recently, TUMS-SPH has started to offer modular teaching (physical attendance of 2 weeks required during each semester), so as to facilitate the process of learning and education while working. [103, 104]. Modifying distance learning pedagogies, for instance by making it text-based to accommodate difficulties accessing internet or incorporating face-to-face course options to minimize isolation, can improve the student learning experience [56, 76, 88, 105]. Similar experiential learning in full-time graduate programs can be accomplished through practicum placements in local communities [44, 106].

*Research* and innovation is essential to adapting existing knowledge and generating new knowledge that addresses population and health system issues [46, 50, 65]. It is becoming a core undertaking of SPHs in LMICs [6, 44, 48, 49, 107]. The Council on Health Research for Development (COHRED) recognizes research and innovation as a key goal of the post-2015 Development Agenda for its contributions to economic prosperity, poverty reduction, and sustainable development [108]. In public health education, research training built into curricula can strengthen comprehension of public health issues by facilitating ‘hands on’ learning for students who are supported by faculty mentorship [6, 60, 64, 84, 108]. For this reason, SPHs in LMICs have adopted multidisciplinary and interdisciplinary approaches in which researchers work collaboratively towards common goals from their distinct or integrated disciplinary perspectives [48, 49, 51, 82]. The intentional multidisciplinary makeup of public health makes SPHs an ideal hub for collaborative practice [6].

Researchers have significant worth in policymaking forums because they can contribute evidence-based recommendations to policy formulation, modification, implementation, evaluation, and promote awareness of under-represented health issues [52, 109, 110]. Knowledge generation through health and related development research conducted by SPHs in LMICs can empower self-sufficiency for decision-making on policy, prevention and treatment strategies, and resource allocation [76]. These benefits translate to increased confidence and autonomy, particularly when negotiating conditions of funding assistance with foreign donors. SPHs can play a significant role in setting the research agenda which otherwise seems complicated due to differences of opinions existing at various levels i.e. society, national, regional and international assemblies [50, 72, 74].

SPHs in LMICs can engage in and lead relevant research through three key vantage points. SPHs in LMICs have a body of faculty and students that is well versed with the local, national, and regional health issues, their determinants and the health priorities [48, 49, 73, 111]. With this expertise, SPHs in LMICs have the ability to collaborate with the relevant stakeholders, i.e. communities, public and private health and development sector organizations, policymakers, and other university departments and schools of public health [46, 64, 111]. A successful experience is that of the National Institute of Public Health (NIPH) in Mexico [112], which leveraged ties with major stakeholders, like the federal Ministry of Health, to facilitate the role of research findings in evidence informed policymaking. A national nutrition survey led by NIPH, as an example, found that Mexican children had high rates of iron deficiency anemia, which after consultation with the Ministries of Health and Social Development, led to inception of programs addressing these specific deficiencies among low-income communities. Another example is that of a university-service partnership for developing effective monitoring systems for ARV rollout in South Africa. This partnership challenged ideologically-driven national policy using scientific evidence thereby building a strong civil society partnership in order to improve provision of HIV care [113, 114].

SPHs in LMICs are in a unique position to lead contextually relevant IR focusing on health policy and systems [69, 115]. Given the importance of context, the involvement of policymakers is critical in IR to ensure that the research is conducted with all relevant stakeholders on-board and the know-do gap is minimized [74, 95, 107, 110, 115]. The National Institute of Health Research (NIHR) in Iran is a dual affiliated (TUMS and MOHME) body responsible for dissemination of national HSR priorities as well as serving as an observatory of the health system. With a prevention and population health focus as well as health system expertise, SPHs in LMICs can move beyond knowledge generation to implementation by fostering collaboration with health system stakeholders [73]. The Higher Education Alliance for Leadership Through Health (HEALTH) Africa Hub is a network of seven SPHs in East and Central Africa that recognized the importance of building HSR capacity in the region through exchange of knowledge and ideas that facilitate the translation of research to national policy [115, 116]. A similar network of 11 African and European universities under the Consortium for Health Policy and Systems Analysis in Africa (CHEPSAA) conducted a review of seven SPHs in Africa. The review found that different levels of assets for health policy and systems research and analysis (HPSRA), require selected interventions [117].

Department of Global Health and Public Policy at SPH-TUMS in Iran has begun collaboration with the School of Population and Health at the University College London (UCL) in the UK to conduct research and education for the interpretation of health related SDGs in Iran and the region. Recently, SPH-TUMS hosted the 1^st^ International Conference on Sustainable Health Development, (24–25 April 2016) in Tehran - Iran [118], where representatives of SPHs from across HICs and LMICs discussed the role of SPHs with regard to education, research and service.

The Committee on Educating Public Health Professionals for the 21st Century to improve health has highlighted multifaceted role of SPHs with responsibilities outlined as [8, 119]: a) educate the educators, practitioners, and researchers; b) serve as the focal point for collaborative research; c) contribute to policy; d) assure quality of public health content in other professional schools; e) provide lifelong learning for the public health workforce to provide healthcare, rehabilitation and palliative services; f) engage actively with communities; and g) provide technical support for population health functions in government and other agencies. These responsibilities illustrate the potential influence of SPHs in communities, institutions, healthcare and policy domains. For example, the JC School of Public Health and Primary Care at the Chinese University of Hong Kong contributes to the above functions by offering both undergraduate (bachelor courses in public health and community health sciences) and postgraduate education (postgraduate diploma and master courses) in public health. It thus acts as a hub for collaborative research and contributes to policy making through consultancy projects from the government and other health organizations [44]. Similarly, National Institute of Public Health in Japan (NIPH) promotes implementation of health policies by fulfilling the training needs of government employees and performing research for the same purpose [62].

In addition to capacity building for health human resources and generating relevant knowledge, SPHs in LMICs can build institutional capacities, develop national and regional health research systems, and engage in regional and global debates on public health issues through inter-sectoral collaborations and *multi-level partnerships* [54, 60, 71, 72, 81, 82, 102, 107, 120, 121]. SPHs in LMICs are positioned to promote health by advocating for neglected health issues, the human rights of marginalized and oppressed groups, and against policies of governments and international agencies that threaten to perpetuate health inequity [64, 90, 107, 120, 122, 123].

SPHs in LMICs can link with fellow SPHs and/or research institutions, civil society groups, private agencies, and professional institutes to pool resources and exchange expertise and experiences in education, research, and informing policy and programs [60, 81, 110]. The Eastern Mediterranean Regional Academic Institutions’ Network (EMRAIN) for public health, the Association of Schools of Public Health in Africa (ASPHA), and the Asia-Pacific Academic Consortium for Public Health (APACPH) are but three examples [52, 63, 80, 120, 121, 124]. Such networks also allow the sharing of experiences in teaching, practicing, and researching public health in different political, economic, and social contexts.

Building on their national presence and regional networking, SPHs in LMICs should not shy away from global partnerships which carry the potential to improve health through knowledge sharing, capacity building, and enhanced research quality [78]. The sustained partnership between the Makerere University Kampala SPH and the Karolinska Institutet (KI), Sweden illustrates a relationship that enables post-graduate education and research [125]. In their joint PhD ‘sandwich’ program, students from Makerere University, who are based primarily in their home country for research, travel to KI for some coursework and supervision support. The Global Network for Women's and Children's Health Research (GNRU) project at CHS-AKU is an example of such partnership between seven countries including United States and those from Latin America, Asia and Africa [126].

The Field Building Leadership Initiative (FBLI) coordinated by the Hanoi School of public health is yet another example of a multi-level, inter-sectoral partnership that aims to advance Eco-health in Southeast Asia (SEA) by addressing the health consequences of agricultural intensification [127]. The FBLI receives funding from the International Development Research Centre (IDRC) and is spearheaded by six academic and research institutions (including SPHs) in China, Indonesia, Thailand, and Vietnam as well as Veterinarians without Borders [128]. Also worth mentioning is the institutional grant awarded by the IDRC to the Faculty of Health Sciences at AUB which supports centers for research, knowledge translation, and practice and offers flexibility in setting of research priorities [129].

Recognizing that communities can have common or conflicting identities that may differ from those assigned by outsiders [123], SPHs in LMICs are in a unique position to forge community partnerships to inform education and research. Such partnerships also provide students with opportunities to utilize knowledge in research and practice [130, 131]. For example, the Urban Health Program of CHS-AKU is a unique model of 25 years of partnership between campus and communities in squatter settlements of Karachi depicting a Primary Health Care prototype with elements of community oriented education and research [131, 132].

Building institutional commitment to knowledge translation within SPHs is also essential, such as in the cases of the Knowledge to Policy (K2P) Center at the Faculty of Health Sciences at the American University of Beirut and the Center for Knowledge Translation and Exchange (KTE) at Tehran University for Medical Sciences [129, 133]. Both Centers are home grown and are actively contributing to the understanding of policymaking in LMICs and to exploring options of how research-driven and tacit local knowledge can inform policy under different political and economic settings. It is also worth mentioning the role of Alliance for Health Policy Systems Research (AHPSR) and Evidence Informed Policy Network (EVIPNET) at WHO in promoting country level partnerships between policy makers, researchers and civil society [134, 135].

In short the SDGs as the global agenda for health by 2030, have created a wide and comprehensive platform for SPHs to develop more meaningful multi-sectoral collaboration [136].

## Conclusion

SPHs can contribute to overcoming several Public Health challenges being faced by LMICs. Most importantly they can develop cadres of competent and well-motivated public health workforce; educators, practitioners and researchers who ask questions that address fundamental health determinants, seek solutions as agents of change within their mandates, provide specific services and serve as advocates for multilevel partnerships. Funding support, human resources, and agency are unfortunately often limited or curtailed in LMICs, and this requires collaboration and constructive partnerships between LMICs and counterpart institutions from HICs. Regional collaborations and global partnerships founded on equal voice and genuine concern about improving the health and wellbeing of LMIC population, including the poor and marginalized, can break the cycle of structural disadvantage that threatens to further health disparities and related human suffering by widening the gap between HICs and LMICs and between rich and poor in LMICs. The platform of SDGs has created an evidence-based forum to address some of the key issues that impede development of SPHs in LMICs.

## Abbreviations

AKU: Aga Khan University
APACH: Asia-Pacific Academic Consortium for Public Health
ASPHA: Association of Schools of Public Health in Africa
CHEPSAA: Consortium for Health Policy and Systems Analysis in Africa
CHS: Community Health Sciences
EMRAIN: Eastern Mediterranean Regional Academic Institutions’ Network
FBLI: Field Building Leadership Initiative
HEALTH: Higher Education Alliance for Leadership through Health
HIC: High-income country
HPSRA: Heath policy and systems research and analysis
IDP: Internally displaced person
IR: Implementation research
K2P: Knowledge to policy center
KI: Karolinska Institute
KTE: Center for Knowledge Translation and Exchange
LMIC: Low and middle-income country
MPH: Master of Public Health
MSc: Master of Science
NCDs: Non-communicable diseases
NIPH: National Institute of Public Health
PHC: Primary Health Care
SDGs: Sustainable Development Goals
SPH: School of Public Health
SPHs: Schools of Public Health
TUMS: Tehran University of Medical Sciences
WHO: World Health Organization
